# The Uses of Pain

**Published:** 1879-02

**Authors:** 


### ARTICLE VIII.

### *The " Uses" of Pain.*

The question is often asked: " What is the use of pain? It is scarcely conceivable that the infliction has no object." There are obviously two aspects of this question; in one, science has an immediate interest; with the other, it has a secondary, but not unimportant, concern. The first is essentially physical. What useful purpose does pain subserve in the animal economy? The answer is thrust upon us by daily observation and experience. There are two sentinels posted, so to say, about the organism, to protect it alike from the assaults of enemies without and exacting friends within. The first of these guardians is the sense of *fatigue.* When this speaks there is need of rest for repair. If the monitor be unheeded, exhaustion may supervene, or, before that point of injury is reached, the second guardian will perhaps interpose for the vital protection—namely, *pain.* The sense of pain, however, is more directly significant of injury to structure, active or threatend, than an excessive strain on function, although in the case of the vital organs, pain occurs whenever the pressure is great. Speaking generally, it may be set down as an axiom that, whatever collateral uses pain may subserve, its chief and most obvious service to humanity is as a deterrent and warning sensation to ward off danger. It is worthy of note, though sufficiently familiar to medical observers, that the absence of this subjective symptom in cases of severe injury is too often indicative of an injury beyond repair. The extinction of pain is not the highest, although it may be a generous impulse. If there were no guardian sensibility of this nature, it would be impossible to live long in the world without self-inflicting the most formidable injuries. That pain, in the second place, has an educational value, as regards the mind and temper, no one can doubt. Some forms of pain would seem to be chiefly intended for this purpose; but even in this view pain has a practical interest, because the higher development of the mind which controls the body, and of which the brain is the formative organ, is a process of physico-mental interest governed by natural laws of which science is perfectly competent to take cognizance.—*London Lancet.*

